# Chronic Patellar Tendon Reconstruction Using Ipsilateral Peroneus Longus Autograft

**DOI:** 10.1002/atn2.70007

**Published:** 2026-05-25

**Authors:** Daniel Vélez‐Díaz, María Camila Botero‐Ossa, Giusseppe Aguado Gómez, Camilo Martínez‐Aguado, Juan David Parra‐Hernández

**Affiliations:** ^1^ Pontificia Universidad Javeriana Cali Department of Orthopedics and Traumatology, Clínica Imbanaco Cali Colombia; ^2^ Fundación Universitaria San Martín Antioquia Colombia; ^3^ Clínica Imbanaco Cali Colombia

## Abstract

Chronic patellar tendon rupture is a rare and challenging injury that compromises knee extension and overall function. Conventional repair often requires augmentation or reconstruction using autografts, allografts, or synthetic materials, with most techniques involving transpatellar tunnels, which increase the risk of iatrogenic fracture. This technical note describes an approach for chronic patellar tendon repair using an ipsilateral peroneus longus autograft in a figure‐of‐eight configuration, avoiding transpatellar tunnels. Fixation is achieved with suture anchors at the tibial tuberosity and a single transverse tibial tunnel. This method provides robust biomechanical stability, minimizes complications, and leverages the advantages of the peroneus longus graft, including consistent size and low donor‐site morbidity.

## Graphical abstract

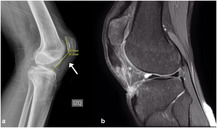

**VIDEO 1:** This surgical demonstration presents a technique for chronic patellar tendon reconstruction using an ipsilateral peroneus longus autograft, avoiding the use transpatellar tunnels. Video content can be viewed at https://doi.org/10.1002/atn2.70007.

Patellar tendon rupture is a rare condition, with a reported incidence of 0.68 per 100,000 individuals.^1^ This rupture compromises the knee extensor mechanism^2^ and causes pain, swelling, and an inability to actively extend the knee.^3^ Patellar tendon ruptures occur more frequently in patients younger than 40 years and are associated with direct or indirect trauma or may result from end‐stage tendinopathy, being more common in men.^4^ Anatomically, most ruptures occur at the proximal insertion at the inferior pole of the patella; mid‐substance tears are less common, and distal avulsion from the tibial tuberosity is extremely rare, occurring in approximately 8% of cases.^5^

Chronic patellar tendon ruptures, defined as injuries older than 6 weeks, involve proximal patellar migration, tendon retraction, scar tissue formation, and altered knee biomechanics.^6^ Surgical repair is the treatment of choice for patellar tendon ruptures. However, in chronic cases, patellar migration, tendon retraction, and quadricep muscle atrophy pose challenges for repair. Therefore, augmentation techniques or tendon reconstruction may be required.^5^, ^6^, ^7^

For repair with augmentation or reconstruction of chronic patellar tendon ruptures, autografts, allografts, or synthetic materials are needed to restore patellar height and extensor mechanism function.^6^, ^7^, ^8^ Although various techniques have been described, autograft reconstruction has shown the best outcomes in chronic injuries, with lower failure rates and fewer complications.^9^

This technical note describes a technique using ipsilateral peroneus longus autograft for augmentation of chronic patellar tendon repair, avoiding transpatellar tunnels and thus reducing the risk of fracture and achieving satisfactory short‐ and mid‐term results.

## SURGICAL TECHNIQUE

### Preoperative Planning

**FIGURE 1:**
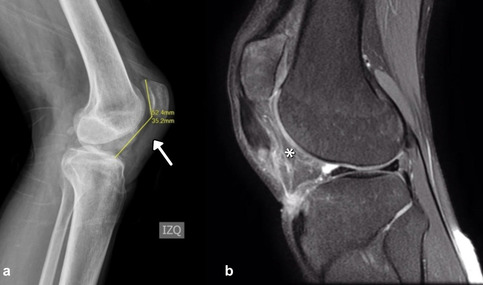
(a) Lateral radiograph at 30° of the left knee showing patella alta (Caton‐Deschamps Index 1.48). (b) MRI (sagittal view) of the left knee confirming patellar tendon rupture (*) without avulsion fractures. (MRI, magnetic resonance imaging.) Position: supine. Operated side: left.

Radiographs assess patellar height using the Caton‐Deschamps index (Figure 1a). Magnetic resonance imaging (MRI) confirms complete patellar tendon rupture at its tibial insertion and rules out additional injuries (Figure 1b).

### Patient Positioning and Preparation

The patient is placed supine with a lateral thigh post for stability. Spinal anesthesia combined with an adductor canal block is administered. A sterile field is prepared; a tourniquet is not used. Two titanium suture anchors (50 mm; LH Rejoin Medical Device, Hangzhou, China) and 2 high‐strength sutures were used (Super sutures; LH Rejoin Medical Device, Hangzhou, China). Step‐by‐step details of the technique are shown in Video 1.

### Exposure

**FIGURE 2:**
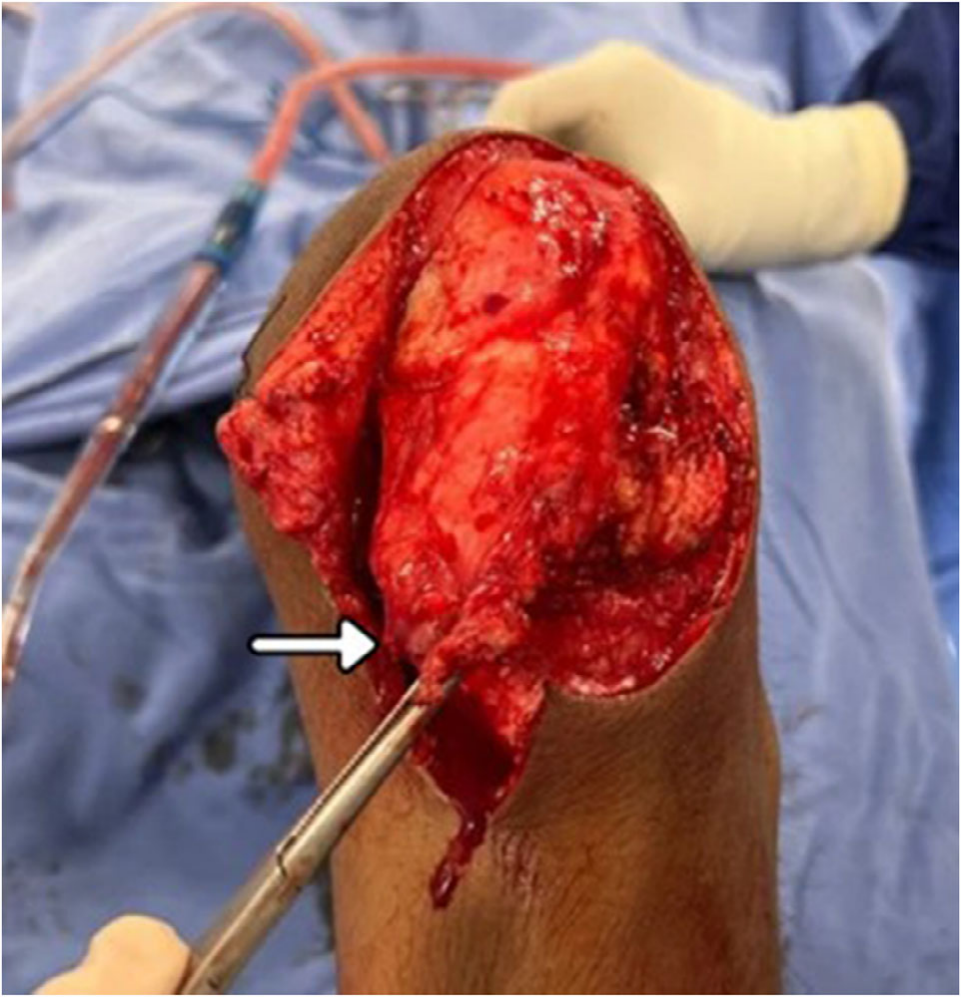
An anterior S‐shaped incision is made over the left knee. Notice completed patellar tendon rupture at its tibial insertion. Scar tissue and non viable tendon edges are excised. Position: supine. Operated side: left.

An anterior S‐shaped incision is made over the knee. Scar tissue and non viable tendon edges are excised (Figure 2). Medial and lateral retinacular releases are performed to allow patellar mobilization and restoration of height.

### Graft Harvesting

**FIGURE 3:**
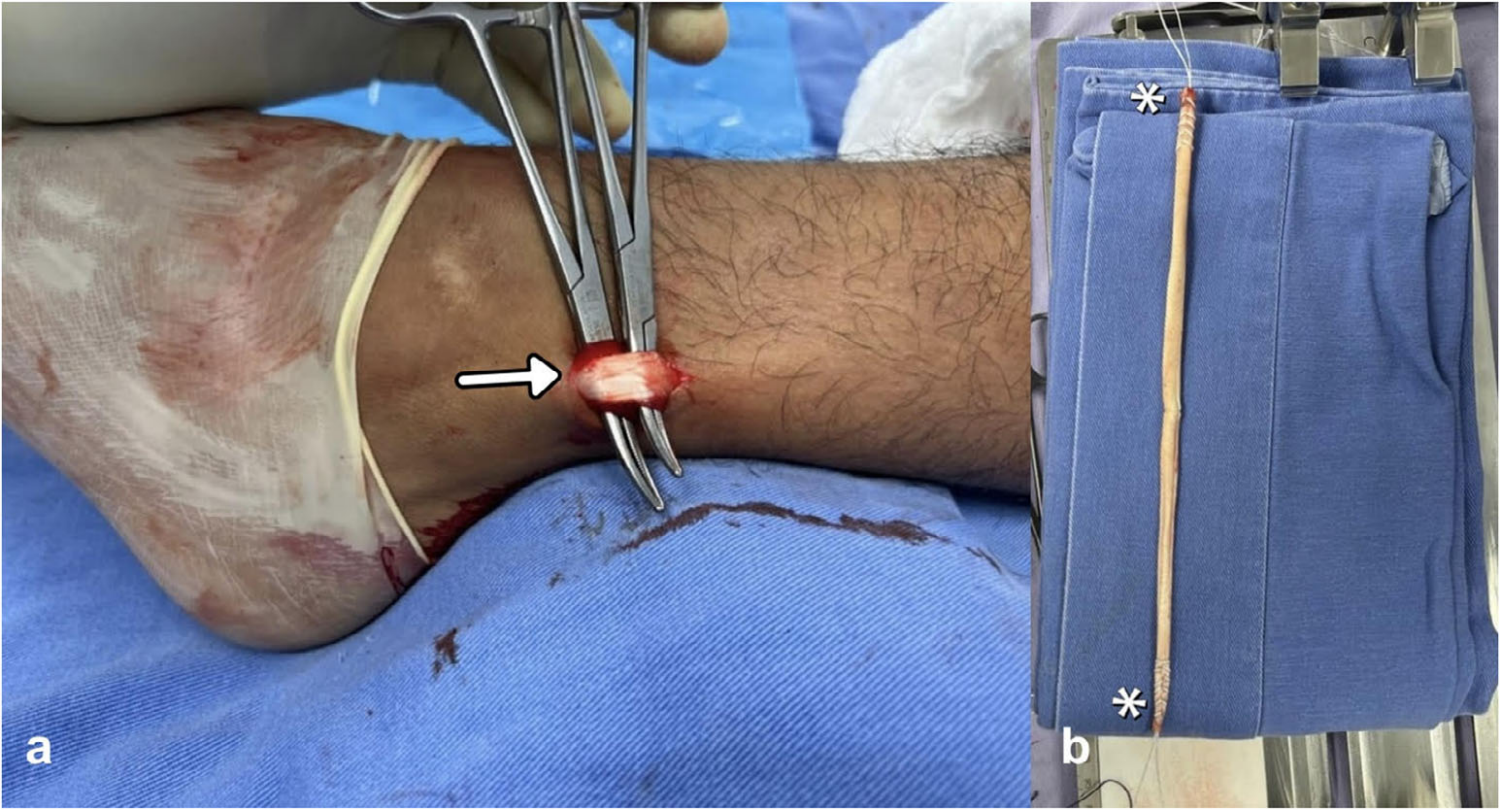
(a) Harvesting of peroneus longus tendon of the left leg (donor site); a 2‐cm incision proximal to the posterior border of the lateral malleolus is performed. (b) The graft ends are whip‐stitched with high‐strength sutures (*), and the graft is prepared on the surgical table. Position: supine. Operated side: left.

A 2‐cm incision proximal to the posterior border of the lateral malleolus is performed. The peroneus longus tendon is identified and harvested after performing a distal tenodesis to the peroneus brevis (Figure 3a). The graft ends are whip‐stitched with high‐strength sutures (Figure 3b).

### Repair and Fixation

**FIGURE 4:**
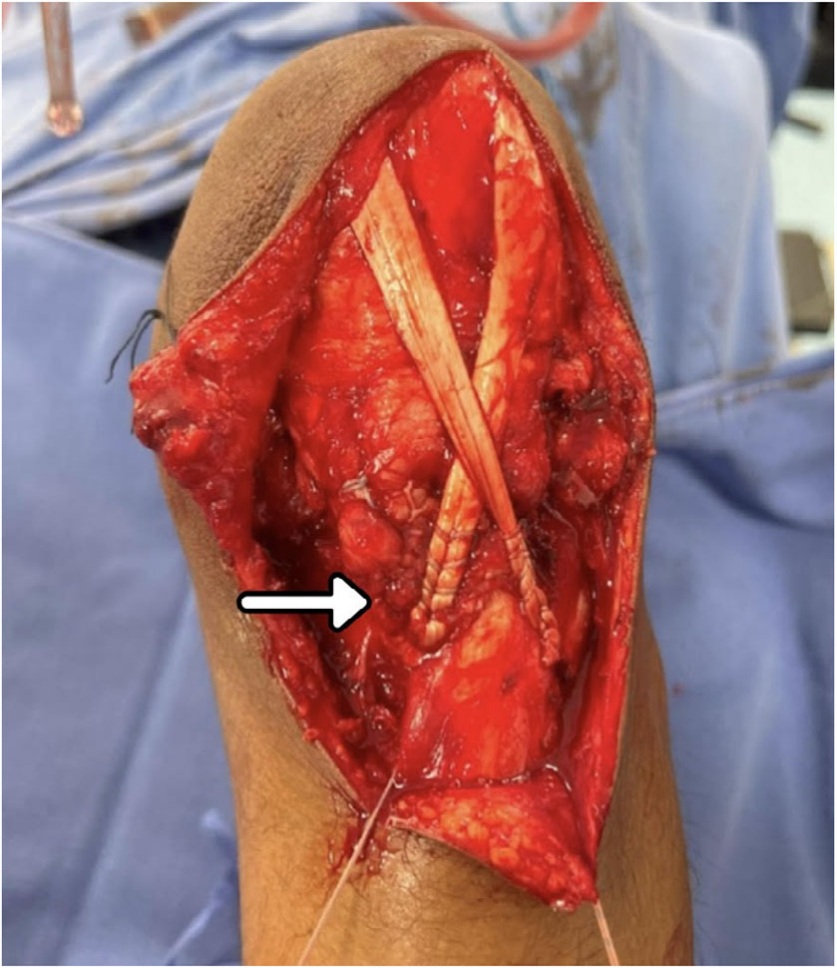
Patellar tendon reconstruction: Anterior aspect of the left knee in a flexed position with graft configured in figure‐of‐eight pattern for augmentation without transpatellar tunnels. Position: supine. Operated side: left.

Two 5.0‐mm titanium suture anchors are inserted at the tibial tuberosity to reattach the native tendon using a Krackow configuration. The peroneus longus autograft is routed in a figure‐of‐eight configuration over the superior pole of the patella and secured through a 5‐mm transverse tibial tunnel, with free ends tied over the medial tibial cortex (Figure 4). Patellar height is restored anatomically.

### Closure and Rehabilitation

**FIGURE 5:**
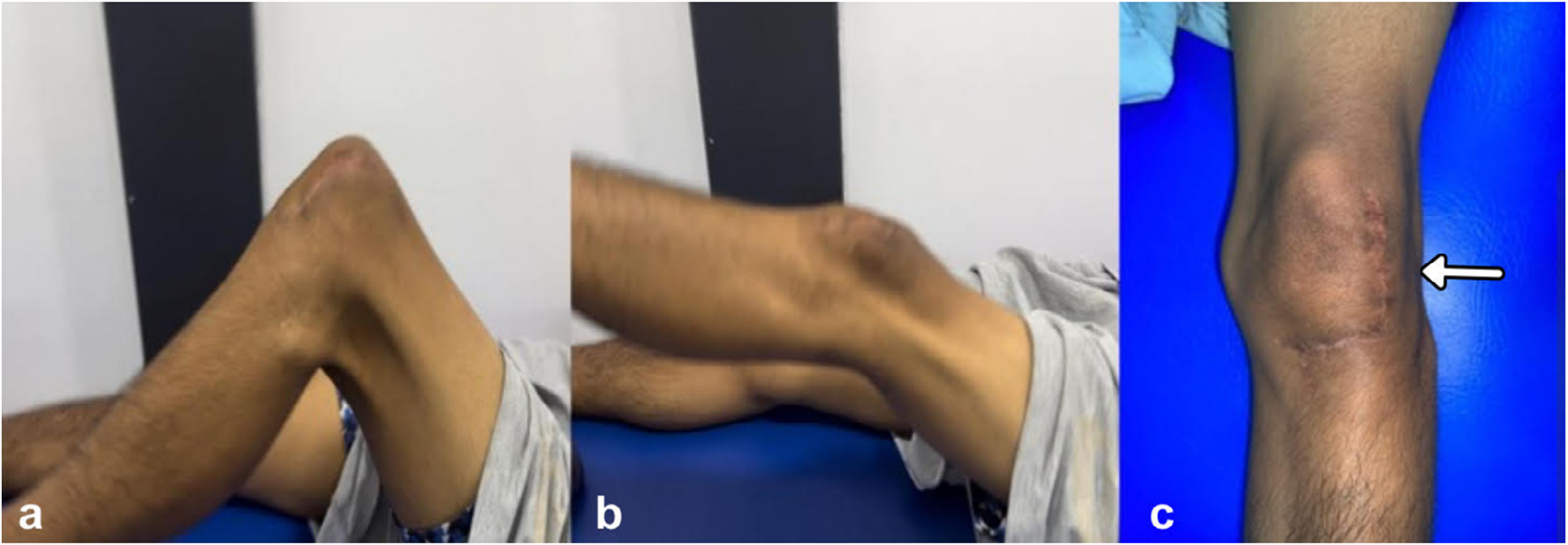
Six‐month follow‐up. (a) Active flexion of the left knee to 120°. (b) Full active extension of the left knee. (c) Well‐healed anterior incision of the left knee. Position: supine. Operated side: left.

Layered closure and retinacular plication are performed. Rehabilitation begins with limited range of motion (0°‐45°) in the first 2 weeks, progressing to 90° by 1 month. Full weight‐bearing and active extension exercises commence after 6 weeks (Figure 5). Written informed consent was obtained from the patient. Pearls and pitfalls of the technique are presented in Table 1.

**TABLE 1:** Technical Pearls and Pitfalls

**Pearls**	**Pitfalls**
Perform complete medial and lateral releases to restore patellar height	Incomplete release → persistent patella alta
Harvest peroneus longus only after distal tenodesis to peroneus brevis	Skipping tenodesis → lateral ankle weakness
Insert anchors precisely at the tibial tuberosity anatomical footprint	Misplacement compromises fixation strength
Tension the graft with the knee in slight flexion to avoid overconstraint	Excessive tension → limited flexion and anterior knee pain

## DISCUSSION

When patellar tendon ruptures occur, associated injuries such as anterior cruciate ligament tears should be ruled out, although they are uncommon.^10^ In this case, MRI confirmed the diagnosis of patellar tendon rupture and excluded associated ligamentous injuries.

Standard treatment for patellar tendon rupture is surgical repair,^11^ which can be performed using bone tunnels or suture anchors.^12^ Among suture techniques, the Krackow technique is widely used as it preserves the vascular supply to the patellar tendon,^11^ and this technique was used in our case to avoid compromising tendon blood supply. When tendon ends can be approximated, repair with augmentation is possible,^13^ as performed in our patient.

Suture anchor repair is a widely accepted alternative to transosseous tunnel fixation for patellar tendon ruptures.^14^, ^15^ Biomechanical studies have shown that this technique results in significantly less cyclic gap formation, which is critical in reducing early repair failure. Although maximum load to failure does not differ significantly between the 2 techniques, suture anchor constructs tend to maintain tendon approximation more effectively under cyclic loading.^14^ Clinically, systematic reviews and comparative studies report a lower re‐rupture rate with suture anchor repair, while reoperation and infection rates remain similar to those observed with transosseous tunnels.^14^, ^15^

In our patient, two 5.0 mm titanium suture anchors were inserted at the tibial tuberosity. High‐strength sutures were passed through the proximal patellar tendon remnant using a Krackow configuration for initial repair and approximation to the tibia. Although suture anchors are associated with higher implant costs,^15^ their advantage in reducing gap formation and improving repair stability supports their use in chronic ruptures, where tendon quality is compromised and early restoration of the extensor mechanism is essential.

Augmentation was necessary in this case to reduce the risk of re‐rupture and improve fixation. Valianatos et al.^16^ described patellar tendon reconstruction using hamstring autografts in 13 patients with 6‐year follow‐up, reporting it as an effective option for chronic patellar tendon injuries without the need for allografts. They showed improvements in Lysholm scores, mean knee flexion of 123°, full extension at 0°, and an Insall‐Salvati index of 1.2, along with quadriceps strength recovery and no reported complications.

Kim et al.^6^ conducted a systematic review evaluating different techniques and implants for chronic patellar tendon reconstruction, including 9 case series with 96 patients. They compared reconstruction techniques using semitendinosus, gracilis, Achilles tendon, or direct repair. Although the semitendinosus was the most frequently used graft, all methods resulted in improved range of motion, extension recovery, quadriceps strength, and patient‐reported outcome measures.

Other techniques and grafts for patellar tendon reconstruction have been described, such as that reported by Patra et al.,^7^ who used a peroneus longus autograft after finding an inadequate semitendinosus tendon during harvest. In their technique, the graft was fixed through two 5‐mm tunnels—one in the patella and one in the tibial tuberosity—passed from lateral to medial through a transpatellar tunnel, and secured in a figure‐of‐eight configuration using a suture anchor at the tibia.

In contrast, our technique also employed a peroneus longus autograft but avoided creating transpatellar tunnels. Instead, the graft was routed over the rectus femoris and vastus intermedius at the superior pole of the patella and secured after passing through a tibial tunnel in an X‐shaped configuration. Avoiding transpatellar tunnels is advantageous because such tunnels increase the risk of patellar fracture, especially in chronic cases with poor bone quality, as highlighted by Pavão.^17^ Preserving the patella's cortical integrity is critical to minimize this complication.

Metallic cerclage constructs, such as the McLaughlin technique, remain an option but are associated with frequent complications, including soft tissue irritation and need for hardware removal.^18^ Our soft‐tissue‐based construct avoids these problems while maintaining robust fixation.

Choosing peroneus longus as an autograft offers several advantages over hamstring tendons. The peroneus longus autograft has consistent length and diameter, reducing the risk of insufficient graft size, a common issue with hamstring tendons, because peroneus longus autografts consistently provide larger graft diameters (mean 8.56 ± 0.93 mm vs 7.44 ± 0.6 mm for hamstrings; *P* < .001).^19^ Also, peroneus longus autograft has low donor site morbidity: A systematic review and meta‐analysis showed that harvesting the peroneus longus tendon results in only minimal impact on ankle function, with small but statistically significant decreases in American Orthopaedic Foot and Ankle Society (AOFAS) score, differences considered clinically irrelevant for daily or athletic activities.^20^ These are extrapolated results of anterior cruciate ligament reconstruction surgery.

Despite its biomechanical advantages and the avoidance of transpatellar tunnels, this technique is not without limitations. First, the procedure requires surgical expertise in both tendon harvesting and graft routing, which may not be universally available. Second, although the peroneus longus autograft shows favorable characteristics—including consistent size and low morbidity—recent studies have reported subtle donor‐site complications. Gök et al. observed that, although the mean ankle AOFAS and Foot and Ankle Disability Index scores were clinically excellent, they were significantly lower compared with the contralateral ankle, and mild paresthesia, dysesthesia, or localized pain occurred in a small subset of patients (3.8%) during early recovery.^19^ Third, in cases of extensive tendon degeneration or severe patella alta, the graft length may be insufficient, necessitating alternative reconstruction strategies. Finally, although suture anchors improve fixation and reduce gap formation, they are associated with higher implant costs and may not be suitable in resource‐limited settings. Further studies with larger sample sizes and longer follow‐up are warranted to assess the long‐term outcomes and complication profile of this technique. Advantages and disadvantages of this technique are presented in Table 2.

**TABLE 2:** Advantages and Disadvantages

**Advantages**	**Disadvantages**
Avoids transpatellar tunnels, reducing fracture risk	Requires surgical expertise in graft harvesting and fixation
Preserves patellar cortical integrity	Limited graft length may be insufficient in severe patella alta
Provides robust biomechanical stability with figure‐of‐eight configuration	Use of suture anchors increases implant cost
Peroneus longus graft has consistent diameter and low donor‐site morbidity	Theoretical risk of ankle weakness in high‐demand patients
Allows early rehabilitation by minimizing construct elongation	Long‐term outcomes in chronic patellar ruptures with this method are limited

In conclusion, our described technique of chronic patellar tendon rupture repair with peroneus longus autograft augmentation emerges as an effective surgical alternative for these patients, offering the advantage of avoiding patellar fracture risk by eliminating the need for transpatellar tunnels. Prospective studies with longer follow‐up and comparisons of different grafts and reconstruction techniques are needed to guide future management.

## DISCLOSURES

The authors (D.V‐D., M.C.B‐O., G.A.G., C.M‐A., J.D.P‐H.) declare that they have no known competing financial interests or personal relationships that could have appeared to influence the work reported in this paper.
